# Partner HIV serostatus disclosure and determinants of serodiscordance among prevention of mother to child transmission clients in Nigeria

**DOI:** 10.1186/s12889-015-2155-x

**Published:** 2015-08-28

**Authors:** Amobi Andrew Onovo, Iboro Ekpo Nta, Aaron Anyebe Onah, Chukwuemeka Arinze Okolo, Ahmad Aliyu, Patrick Dakum, Akinyemi Olumuyiwa Atobatele, Pamela Gado

**Affiliations:** Department of Public Health, University of Calabar, Calabar, Cross Rivers Nigeria; Department of Health Systems Strengthening, Institute of Human Virology, Nigeria (IHVN), Abuja, FCT Nigeria; Strategic Information, Institute of Human Virology, Nigeria (IHVN), Abuja, FCT Nigeria; Office of the CEO, Institute of Human Virology, Nigeria (IHVN), Abuja, FCT Nigeria; United States Agency for International Development (USAID), Abuja, FCT Nigeria; A39 Lazarus Mouka Crescent, El-salem Estate Lugbe, Abuja, 900286 FCT Nigeria

## Abstract

**Background:**

Serodiscordance exists when the known HIV result of one member of a couple pair is positive while that of his/her partner is negative. In sub-Saharan Africa, in stable long-term couple partnerships (married or cohabiting), serodiscordance is a growing source of HIV-transmissions. This study aimed to ascertain across Nigeria, serodiscordance prevalence, partner HIV status disclosure and explore associations between suspected determinants and serodiscordance among PMTCT enrolled HIV positive pregnant women and their partners.

**Methods:**

A retrospective Quality of Care performance evaluation was conducted in July 2013 among 544 HIV positive pregnant enrolees of PMTCT services in 62 comprehensive facilities across 5 of Nigeria’s 6 geo-political zones. Data of client-partner pairs were abstracted from pre-existing medical records and analysed using chi-square statistics and logistic regression.

**Results:**

A total of 544 (22 %) of 2499 clients with complete partner details were analysed. Clients’ age ranged from 15 to 50 years with a mean of 30 years. Serodiscordant prevalence was 52 % and chi-square test suggests no significant difference between serodiscordant and seroconcordant clients and their partners (*p* = 0.265). Serodiscordant rates were closely associated trend wise with national HIV sero-prevalence rates and the median CD4+ count was 425 ul/mm^3^ (IQR: 290–606 ul/mm^3^). Similar proportion of clients (99 %) received testing and agreed to disclose status to their partners. Yet, there was no association between clients agreement to disclose HIV status to their partners and these partners getting tested and receiving results (*p* = 0.919). Significantly, 87 % of clients in concordant HIV positive relationships appeared to be symptomatic (WHO clinical stage 3 or 4) compared to 13 % clients in HIV-discordant relationships (*p* < 0.003). Client’s age and CD4+ count did not aptly predict serodiscordance (Wald = 0.011 and 0.436 respectively). However, the WHO clinical staging appeared to be a better predictor of serodiscordance and concordance than other variables (Wald = 3.167).

**Conclusions:**

The results suggest that clinical staging (WHO) could be a better predictor of client- partner pair discordant or concordant HIV serostatus. Early partner testing and notification can avert seroconversion, hence properly designed and mainstreamed interventions that target serodiscordant couples are essential.

**Electronic supplementary material:**

The online version of this article (doi:10.1186/s12889-015-2155-x) contains supplementary material, which is available to authorized users.

## Background

Prevention of mother to child transmission (PMTCT) is vital for HIV/AIDS control [1–3]. In developing countries, antiretroviral (ARV) prophylaxis averted HIV infection in 409,000 children between 2009 to 2011 [4]. However, PMTCT is not optimum in Nigeria, with largest number of new paediatric HIV infection globally occurring here – averaging 60 000 yearly from 2009 to 2012 [5]. For enhanced PMTCT, mutual knowledge of partner’s HIV status is ideal [6], yet clinicians frequently have to presume that HIV-positive women have partners whose status are either negative or unknown.

We introduce the term ‘serodiscordancy’ to encompass serodiscordant and seroconcordant heterosexual couples. Serodiscordant or serodiscordance applies when one partner of an intimate couple pair’s HIV result is positive and other negative. If positive HIV results are known for both, this is a seroconcordant or seroconcordance relationship [7]. A discordant status is particularly risky because regular coitus without condoms is more probable in stable long-term partnerships (married or cohabiting).

Serodiscordance is documented as a growing source of HIV-transmissions in sub-Saharan Africa (SSA) [8, 9], thus averting intra-couple transmission could amply impact the epidemic [10]. Proper condom use [11] diminishes exposure and early ARV initiation lowers viral replication which limits transmission of human immunodeficiency virus type 1 (HIV-1) in serodiscordant couples [12, 13].

Unprecedented antiretroviral therapy scale up has improved access for millions worldwide. In Nigeria, this means rapidly expanding numbers of People Living With HIV (PLWHIV) enrolled in treatment programs [14]. For PLWHIV, evaluating the effectiveness of existing programs allows for development of context- specific strategies that addresses their sexual/reproductive aspirations, and attendant risks [15], including participation in a HIV serodiscordant relationship.

Furthermore, in stigma-rife parts of SSA, gender directed abuse may accompany disclosure of a HIV positive result [16, 17]. Though aberrant social-cultural and gender power constructs are plausible explanations, the availability of sexually transmitted infections (STIs) control services including pre-exposure prophylaxis must be considered

Several studies in Nigeria have examined serodiscordance and disclosure [18–20], but these were largely geographically-limited. From select PMTCT treatment facilities across Nigeria, this paper explores associations between certain predictors and serodiscordance among pregnant HIV-positive clients and their partners.

## Methods

### Location

Nigeria is organized into six geo-political zones, which encompass neighbouring states (see Additional file 1). The study was conducted in 5 of the 6 geo-political zones excluding the North East zone due to security challenges. In July, 2013, locations providing comprehensive HIV/AIDS treatment services to pregnant women and their partners for at least one year were enrolled. Eventually, 62 ARV (known as comprehensive service delivery points) spanning primary, secondary and tertiary level facilities were eligible.

### Population

All pregnant women aged ≥ 15 years enrolled in PMTCT program in selected facilities in the study locations. Based on National PMTCT guidelines, pregnancy in the HIV positive woman is an indication for ARVs irrespective of CD4, VL or clinical stage [21].

### Design

A retrospective performance evaluation that measures Quality of Care (QoC) provided to HIV positive pregnant women and their exposed infants, particularly in the children’s first 2 years of life, in a 6-month review period (January 1^st^ – June 30^th^, 2013).

### Sampling technique

The QoC evaluation exercise included four (4) different categories of women (inclusion criteria) which were:

i. HIV positive pregnant women identified in Antenatal Care (ANC) in 6 months prior to the beginning of the review period (July – December, 2012).
ii. Unbooked HIV positive pregnant women who delivered at the facility in 6 months prior to the beginning of the review period (July – December, 2012).
iii. All deliveries by HIV positive pregnant women (booked and unbooked) during 6 months of the review period (January 1^st^ – June 30^th^, 2013).
iv. HIV positive pregnant women who delivered in the facility 12 – 18 months prior to the beginning of the review period (July 2011 – December 2012).

Unbooked women are those who appear for the first time in the facility when in labour.

Sample frame was developed by applying the four inclusion criteria stated above to each audited patient’s medical folders. The sampling method varied from site to site, depending largely on the site’s information management system. For Electronic Medical Record enabled sites (which includes patient IDs, up-to-date clinical and other follow up information) all eligible sample of PMTCT patients was generated and audited. For paper-based medical record sites, a *systematic random* sampling was used to generate the required sample of PMTCT patients audited.

### Sample size

This was determined for each of the different sampling methods applied for the two categories of sites mentioned above using the *HIVQUAL* [22] sampling methodology (see Additional file 2). These relied on the following 5 simple steps;

1. Determine the number of HIV+ pregnant women in each category (i – iv) in the 6 months reference period for each of categories i – iv (X_i_ - X_iv_).
2. Determine PMTCT sample size for each of categories i - iv (Y_i_ – Y _iv_) from the HIVQUAL sample size determination table (see Additional file 2).
3. Input the Y (Y_i_ – Y _iv_) number of ANC IDs on the Random Number List(RNL).
4. Divide the total number of HIV+ pregnant women in each category (X_i_ - X_iv_) by (Y_i_ – Y _iv_) respectively. Approximate (X/Y) to the nearest whole number N.
5. Identify every Nth HIV+ woman’s ID, impute them serially into the RNL forms. Quality of PMTCT care will be reviewed for women with these IDs.

Where ‘’X” is the total sample of eligible population (Sample Frame) and ‘’Y” the sample size generated from HIVQual sample size determination table.

### Data collection

The QoC performance evaluation process involved use of care and treatment service delivery indicators and entries in national data collection tools (DCTs) to guide data abstraction from randomly selected pre-existing patients’ medical records. The four inclusion criteria stated in section 2.4 were applied to each audited medical folders.

For the PMTCT program component of the exercise, HIV negative pregnant women and HIV positive women with incomplete partner data were excluded from the sample of folders audited. Reliable and site specific data from the following registers - the Antenatal Care (ANC), HIV counselling and Testing, Delivery, Maternal, Partner, Early Infant Diagnosis, Child follow-up and ARV were reviewed and used to determine the pool of eligible sample of patients audited. The inclusion and exclusion criteria were applied on each step of the performance evaluation audit.

### Data analysis

Data was sorted, coded, keyed into Microsoft Access database and exported to SPSS 20.0 for analysis. Results were presented using charts, graphs and frequency tables. Descriptive statistics such as mean, frequencies and percentages was used to describe and summarize findings. Contingency tables were developed, logistic regression and chi-square statistics tested associations between variables and level of significance (α = 0.05).

### Ethical consideration

Ethical approval was obtained in line with the standard ethical procedures from the United States Centers for Disease Control and Prevention (CDC). The National Research Health Ethics Committee (NRHEC) of the Federal Ministry of Health Nigeria approved the primary survey. No clients were recruited for the PMTCT performance evaluation.

Study abstraction forms collated data from pre-existing client medical records and facility registers. Within facilities, abstraction was performed only in secure areas and data with or containing links to unique identifiers of study subject were kept under lock and key, until these records were appropriately returned to the facility records sections. All individuals involved in data abstraction and analysis received training on confidentiality procedures and obtained Collaborative Institutional Training Initiative (CITI) human subjects certification.

## Results and discussion

### Descriptive analysis

The records of a total of 2,499 HIV positive women were sampled, but 544 (22 % of the total eligible population) who met inclusion criteria for clients with matched partner details was used in the analysis. Test for associations of the continuous variables (Age, baseline CD4+ count and WHO clinical staging) and dichotomous variables (received HTC, agreement to notify partner i.e., disclosure and clients who received ARV) were explored (Table 1).

**Table 1:** Proportion of total HIV clients by partners

Variables	Frequency	Percent	Chi-Square
Client’s with no partners	1955	78	796.68 (*p* < 0.001)*
Client’s matched with partners	544	22	
Total	2499	100	

*Significant (*P*-value < 0.05)

Approximately 78 % of clients with no available partner information (i.e., no partner HIV status documented in PMTCT partner register) were excluded. Poor documentation of partner HIV status in the PMTCT partner register at the study sites may have impacted on the results of the analysis as evident with the significant Chi-square goodness of fit *χ*^2^ (1) = 796.68, *p* = 0.001.

#### Serodiscordancy characteristics

Approximately 52 % (285) of total client-partner pairs analysed were serodiscordant (i.e., HIV sero-positive pregnant woman with HIV sero-negative partners) while 48 % (259) of same population was seroconcordant (HIV sero-positive pregnant woman with HIV sero-positive partners). No significant difference was observed between clients engaged in serodiscordant relationships or their seroconcordant counterparts, Chi-square goodness of fit *χ*^2^ (1) = 796.68, *p* = 0.265. Low partner involvement and poor site documentation of partner PMTCT data may however account for the lack of difference in the outcome of interest (Table 2).

**Table 2:** Proportion of serodiscordancy among HIV positive pregnant women and their sero-negative partners

Variables	Frequency	Percent	Chi-Square
Seroconcordant clients	259	48	796.68 (0.265)
Serodiscordant clients	285	52	
Total	544	100	

#### Characteristics of geo-political regions

High serodiscordant rates were noted across 3 of the 5 (60 %) study locations. The affected geopolitical regions are the South-South (SS) 60.2 %, North-Central (NC) 51.9 % and South-West (SW) 53.1 %. In contrast, high seroconcordant rates were recorded in the North-West (NW) 83.3 % and South-East (SE) 50.8 %. In general, there was no significant association between belonging to a serodiscordant or seroconcordant relationships with geo-political regions; *χ*^2^ (1) = 6.147, *p* = 0.188 (Table 3).

**Table 3:** Proportion of serodiscordancy by geo-political zones

Variables	Total		Seroconcordant		Serodiscordant		Chi-square (*p*-value)	Symetric Measure	
	n	%	n	%	n	%		Cramer’s V	Phi coefficient
South South	103	100	41	39.8	62	60.2	6.147 (0.188)	.11	
North Central	270	100	130	48.1	140	51.9			
North West	6	100	5	83.3	1	16.7			
South West	32	100	15	46.9	17	53.1			
South East	132	100	67	50.8	65	49.2			
Total	543	100	258	100	285	100			

Significant (*P*-value < 0.05)

**Fig. 1:**
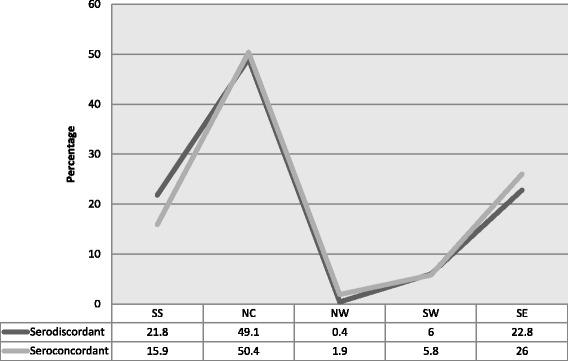
Trend lines showing pattern of serodiscordant and seroconcordant relationships across 5 geo-political regions

The line graphs on prevalence by region shows no distinct separation of clients in seroconcordant and serodiscordant relationships. Peak prevalence rates of seroconcordant (50.4 %) and serodiscordant (49.1 %) clients and their partners were recorded in the North Central region. This is consistent with the 2010 National HIV Sero-prevalence Sentinels Survey (NHSS) outcomes which report the highest HIV prevalence the North Central (7.7 %) region (Fig. 1).

**Fig. 2:**
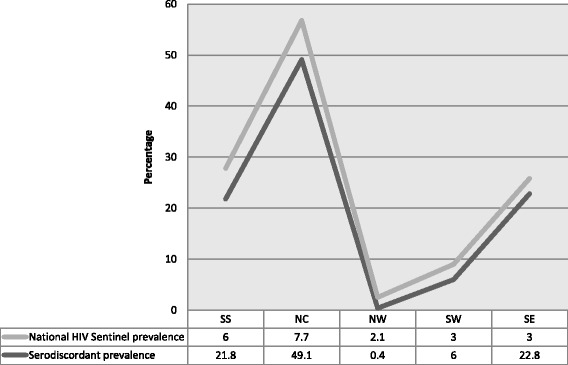
Trend line showing pattern of National ANC HIV Sero-prevalence (NHSS) and serodiscordant prevalence across 5 geopolitical regions

Line graphs of the 2010 NHSS [23] plotted alongside the serodiscordance prevalence from this study across the 5 study regions indicates a glove fit pattern. Highest prevalence was in the NC with NHSS (7.7 %) and serodiscordant prevalence (49.1 %). Lowest prevalence was in NW with NHSS (2.1 %) and serodiscordant prevalence (0.4 %). This correlates suggest that the NHSS could serve as a proxy for estimating prevalence of serodiscordancy among clients and their partners. Thus, the NHSS could guide serodiscordant intervention, extrapolation and or estimating prevalence of Nigeria’s serodiscordancy (Fig. 2).

#### Clinical characteristics

Exactly 55 % of the study clients in seroconcordant relationships demonstrated CD4+ count less than 350 ul/mm^3^ compared to 49 % of clients in serodiscordant relationships who have CD4 count greater than 350 ul/mm^3^. However, there was no association between clients being in seroconcordant relationship and serodiscordant relationships with CD4+ count; *χ*^2^ (1) = 0.143, *p* = 0.706 (Table 4).

**Table 4:** Proportion of clinical characteristics

Variables	Total		Seroconcordant		Serodiscordant		Chi-square (*p*-value)	Symetric measures	
	n	%	n	%	n	%		Cramer’s V	Phi coefficient
Baseline CD4+ count							0.143 (0.706)		0.04
CD4 < 350	40	100	22	55.0	18	45.0			
CD4 > 350	76	100	39	51.3	37	48.7			
WHO clinical staging							8.751 (0.003)^*^		−0.15
Asymptomatic	373	100	178	47.7	195	52.3			
Symptomatic	15	100	13	86.7	2	13.3			

*Significant (*P*-value < 0.05)

In terms of WHO clinical staging, clients in seroconcordant relationships (87 %) appeared symptomatic (i.e., having a WHO clinical stage of 3 or 4) as compared to 52 % of the clients in serodiscordant relationships who were asymptomatic (i.e., having a WHO clinical staging of 1 or 2). The chi-square test suggest that there is a significant but weak association between clients being in seroconcordant or serodiscordant relationships with WHO clinical staging; *χ*^2^ (1) = 8,751, *p* < 0.003, Phi coefficient (φ) = −0.150.

**Fig. 3:**
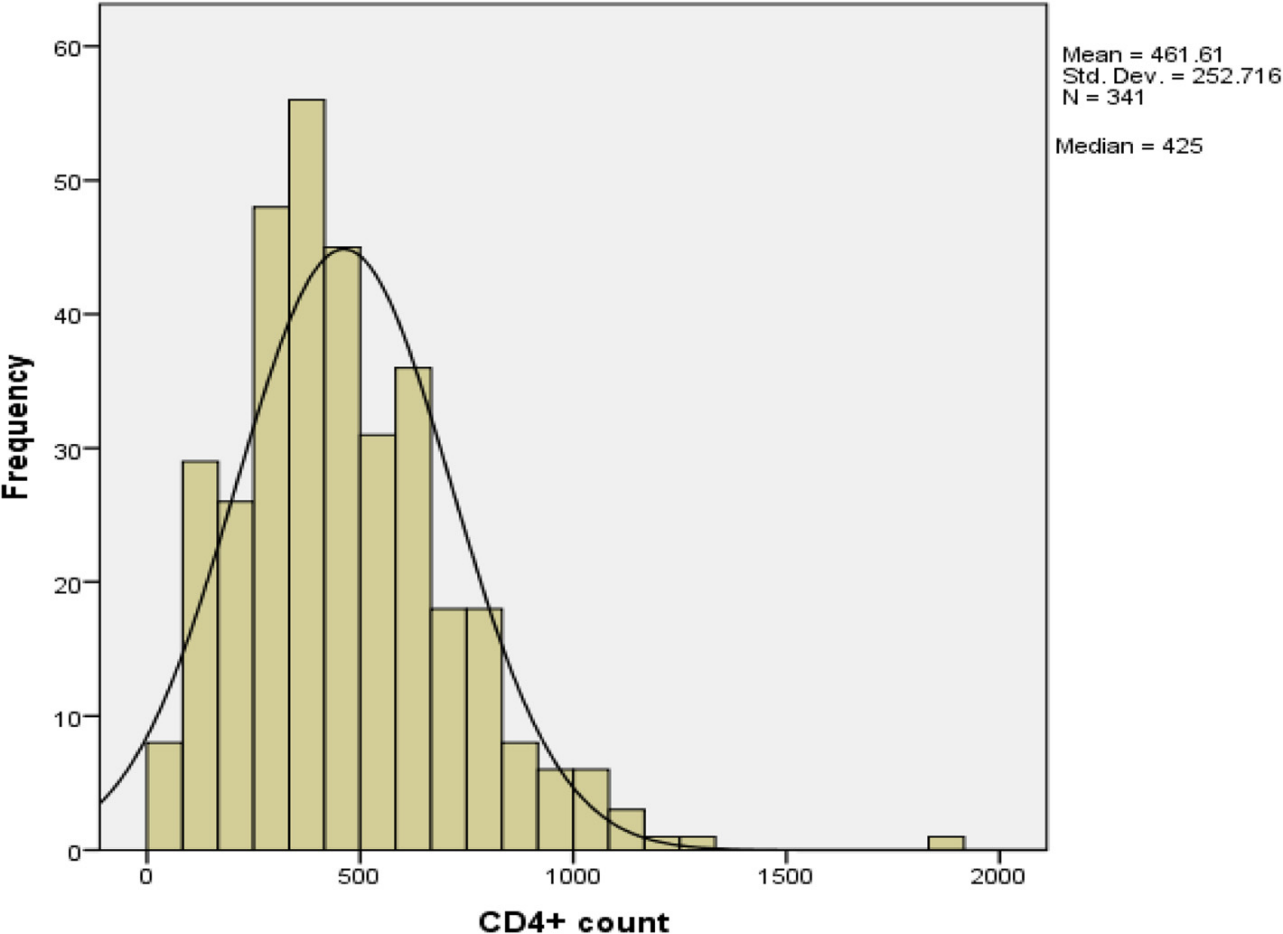
Histogram showing median distribution of client’s total baseline CD4+ count

Baseline CD4+ counts for 63 % (342) of all 544 clients in the study were accounted for. Of this, the median CD4+ count was 425 ul/mm^3^ (IQR: 290–606 ul/mm^3^) (Fig. 3). The CD4+ count distribution displayed a non – normal spread with a Shapiro-Wilk's test = 0.093, *p* < 0.001, which was significant at the 95 % confidence interval (CI). The slope is left leaning (positive) with skew of 1.052 (standard error = 0.132) and kurtosis of 2.426 (standard error = 0.263). A multi country trial among African serodiscordant couples revealed high mortality rates at CD4 counts < 500 cells/μl with 8.9 deaths/1000 person years at CD4 counts of 350–499 cells/μ [24].

**Fig. 4:**
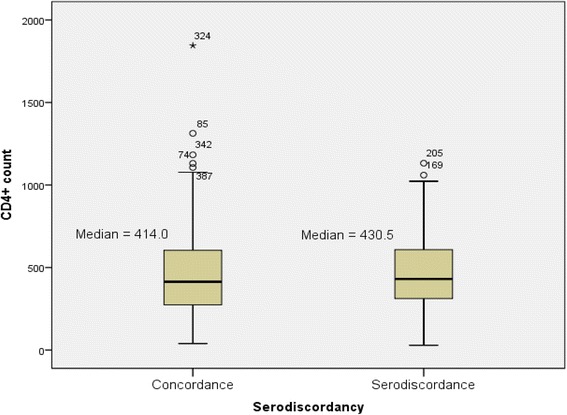
Boxplot showing median distribution of client’s total baseline CD4+ count with clients HIV sero-status

The median baseline CD4+ count among clients in serodiscordant relationship was 430.5 ul/mm^3^ compared to 414 ul/mm^3^ among clients in seroconcordant relationship (Fig. 4). The boxplot displayed a non – normal distribution between the 2 distinct HIV Sero-status with a Shapiro-Wilk’s test = 0.876, *p* < 0.001, which was significant at the 95 % CI.

#### Age demographic and clients HIV disclosure characteristics

**Fig. 5:**
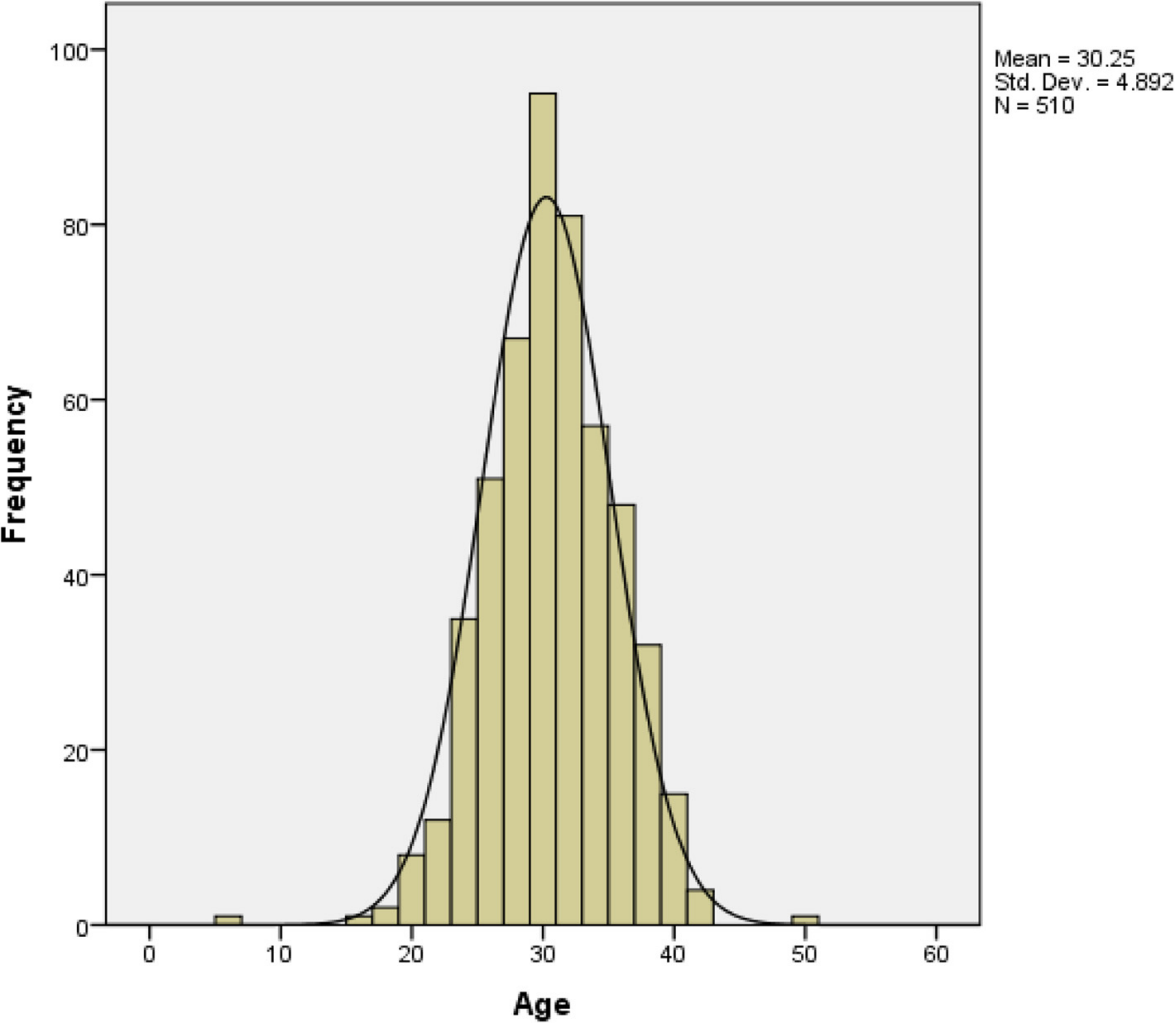
Histogram showing age distribution of study clients

Client’s age distribution demonstrated *Gaussian* properties with an almost exact mean, median and mode. The mean age of the study population was 30.3 ± 4.89 years and ranged from 15 to 50 years (Fig. 5).

**Fig. 6:**
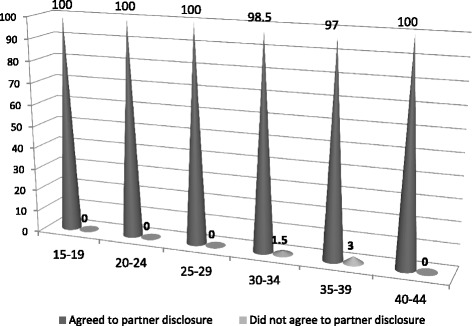
Percent distribution of clients who agreed to partner disclosure by age

In terms of clients age, 80 % of the study clients in seroconcordant relationship belong to the 15–19 years category, making it the highest age group among Seroconcordant clients, while 39 % belong to the 40–44 age category, −the least. Among clients in serodiscordant relationships, 62 % of the clients are in the 40–44 years category- highest, while 20 % belong to the 15–19 age category, − least. The chi-square test of significant suggests no association between clients in seroconcordant relationship and serodiscordant relationships with Age (Fig. 6).

Figure 6 shows the age disaggregated disclosure pattern of clients. As seen across all ages’ majority stated agreement to partner disclosure.

In terms of HIV testing and (HTC), less than two thirds (53 %) of the clients in serodiscordant relationships received HIV testing while 47 % of clients in seroconcordant relationships did not. 54 % of the clients in serodiscordant relationships agreed to disclose their HIV status to their partners, while 46 % of the clients in seroconcordant relationships reported otherwise.

The outcome of HIV testing and counselling (HTC) of PMTCT intervention in this study appeared a better fit among serodiscordant clients and their partners as compared to seroconcordant clients and their partners. The chi-square test of significance suggest no associations between these groups regarding HTC and clients agreement to disclosure of HIV status respectively; *χ*^2^ (1) = 0.012 and 0.024, *p* = 0.911 and 0.877.

#### Clients disclosure of HIV status characteristics

Almost all (99 %) of study clients agreed to disclose their HIV status to their partners. There was a significant difference between clients who agreed to partner disclosure and clients who did not; *χ*^2^ (1) = 374.16, *p* < 0.001. Efficient and effective HIV testing and counselling services implemented at the study sites may account for the differences (Table 5).

**Table 5:** Proportion of partner disclosure among study clients

Variables	Frequency	Percent	Chi-square
Agreed to partner disclosure	386	99	374.16 (*p* < 0.001)*
Did not agree to partner disclosure	4	1	

*Significant (*P*-value < 0.05)

It is evident that across all study regions, there is strong momentum among majority of study clients to disclose their HIV status, however a few of these clients (3 %) in the South- South region reported otherwise. The Pearson chi-square test of association suggests no significant association between clients disclosure of HIV status and study regions; *χ*^2^ (1) = 7.965, *p* = 0.093. To improve objective measures that ensure good HTC outcomes, this result underlies the necessity by health providers to initiate innovative processes to verify that clients who indicated agreement to partner disclosure actually follow through or are supported to achieve this (Table 6).

**Table 6:** Proportion of partner disclosure across the study regions

Variables	Agreed to partner disclosure		Did not agree to partner disclosure		Chi-square (*p*-value)	Symmetric measure	
	n	%	n	%		Cramer's V	Phi coefficient
South -South	94	96.9	3	3.1	7.965 (0.093)	0.14	
North Central	226	100	0	0.0			
North West	4	100	0	0.0			
South West	28	100	0	0.0			
South East	33	97.1	1	2.9			
Total	385	99	4	1.0			

Significant (*P*-value < 0.05)

The cross tabulation statistics output of clients disclosure status was 99.7 % between clients who received HTC, and clients agreed to disclosure of HIV status and suggests high chances or likelihood of partners getting tested and counselled in the PMTCT program. However, the chi-square statistics suggests no associations between these variables; *χ*^2^ (1) = 0.010, *p* = 0.919 (Table 7).

**Table 7:** Proportion of clients who agreed to disclosure of HIV status by their partners who received testing and counselling

Variables	Total		Agreed to disclosure of HIV status		Did not agree to disclosure of HIV status		Chi-square (*p*-value)	Symmetric measures	
	n	%	n	%	n	%		Phi coefficient	Cramer's V
Partners tested and received results							0.010 (0.919)	0.005	
No	4	100	4	100	0	0			
Yes	385	100	384	99.7	1	0.3			

Significant (*P*-value < 0.05)

### Logistic regression

A predictive model, binomial logistic regression was performed to ascertain the effects of Age, CD4+ count and WHO Clinical staging on the likelihood that participants were in concordant or discordant relationships. The logistic regression model was not statistically significant, Chi-Square *χ*2 (4) = 4.012, *p* > 0.05. The model explained 19 % (Nagelkerke R^2^) of the variance in serodiscordancy and only correctly classified 56.3 % of cases. Sensitivity was 94.6 %, specificity was low at 14.1 %, positive predictive value was 54.9 % and negative predictive value was 70.4 %. Client’s age and CD4+ count did not aptly predict serodiscordance (Wald = 0.011 and 0.436 respectively). However, the WHO clinical staging appeared to be a better predictor of serodiscordancy than other explanatory variables (Wald = 3.167) with an odds ratio of 0.234 (95 % CI: 0.047 to 1.159) (Table 8).

**Table 8:** Logistic regression model

Model summary									
Step	−2 Log likelihood	Cox & Snell R Square	Nagelkerke R square						
1	389.005^a^	0.014	0.019						
Hosmer and Lemeshow test									
Step	Chi-square	df	Sig.						
1	5.668	8	0.684						
Classification table									
	Observed		Predicted						
			Serodiscordancy		Percentage correct				
			Concordance	Serodiscordance					
Step 1	HIV Sero-status	Concordance	19	116	14.1				
		Serodiscordance	8	141	94.6				
	Overall Percentage				56.3				
The cut value is .500^b^									
Variables in the equation									
		B	S.E.	Wald	df	Sig.	Exp(B)	95 % C.I.for EXP(B)	
								Lower	Upper
Step 1^a^	WHO_clinical_stage(1)	−1.453	0.816	**3.167**	1	0.075	0.234	0.047	1.159
	CD4+ count	0	0	0.436	1	0.509	1	0.999	1.001
	Age	−0.003	0.024	0.011	1	0.915	0.997	0.951	1.046
	Constant	0.363	0.781	0.216	1	0.642	1.438		

Bold data is the test statistics predicts serodiscordant or concodant partner relationaship, since the wald coefficient 3.167 is greater than 0

^a^Estimation terminated at iteration number 4 because parameter estimates changed by less than .001

^b^Variable(s) entered on step 1: WHO_clinical_stage, CD4_raw, and Age

The disclosure process in the national HCT guidelines neither elicits partners’ age nor enforces enrolment/ARV status of HIV + partners [7] and weren’t available for analysis. The small sample size of the explanatory variable- WHO clinical stage (less than 15 for the WHO symptomatic category) could have affected the reliability of the Sig estimates, as it does not meet minimum statistical recommendations of at least 15 cases.

## Limitations

Data quality likely influenced findings, Nigeria’s health data management systems require urgent improvement. The retrospective methodology restricted research to use of historical data. Consequently, other important variables particularly partner’s age, ARV status/regimen, frequency and route of coitus were not available for analysis.

## Conclusions

In conclusion, the regression model was a poor fit and indicates no significant association between the suspected determinants and serodiscordance. However, the WHO clinical staging appeared to be a better predictor of serodiscordancy than the other explanatory variables. For detailed explanation see logistic regression section above.

Effective disclosure begins at pre testing and runs through post counselling as explained in national guidelines. The algorithm contained is adequate on adhering to confidentiality and ensuring psychosocial support [25]. Urgent attention is required to create, mainstream and harmonize male partner data in relation to pregnant women accessing PMTCT.

Likewise, research is essential on partners of HIV positive males (females in heterosexual partnerships and male partners of Men who have sex with Men-MSM). Associations with viral load, gestational age, nutrition, genetics and parity are other important indices or predictors and should be explored in subsequent studies.

Early partner testing and notification can avert seroconversion, thus expanding/redesigning interventions to accommodate serodiscordant couples is essential. Flexible policies on use of data generated by PEPFAR implementing partners (USAID/CDC) program for serodiscordant studies are helpful.

Serodiscordancy findings should be tracked in the national Mode of Transmission (MOT) reports across all the risk groups stated. Eventually, serodiscordant rates have methodological and statistical implications for relying on ANC HIV sero-prevalence as proxy for estimating national HIV prevalence.

## Additional files

Additional file 1:
**Study regions and state.** (PDF 373 kb) Additional file 2:
**HIVQual sample size determination chart based on 95 % CI.** (PDF 173 kb)
