# Supplementary figures and images for: Partner HIV serostatus disclosure and determinants of serodiscordance among prevention of mother to child transmission clients in Nigeria
Source: BMC Public Health. 2015 Aug 28;15:827. doi: 10.1186/s12889-015-2155-x (PMC4551711; doi:10.1186/s12889-015-2155-x)

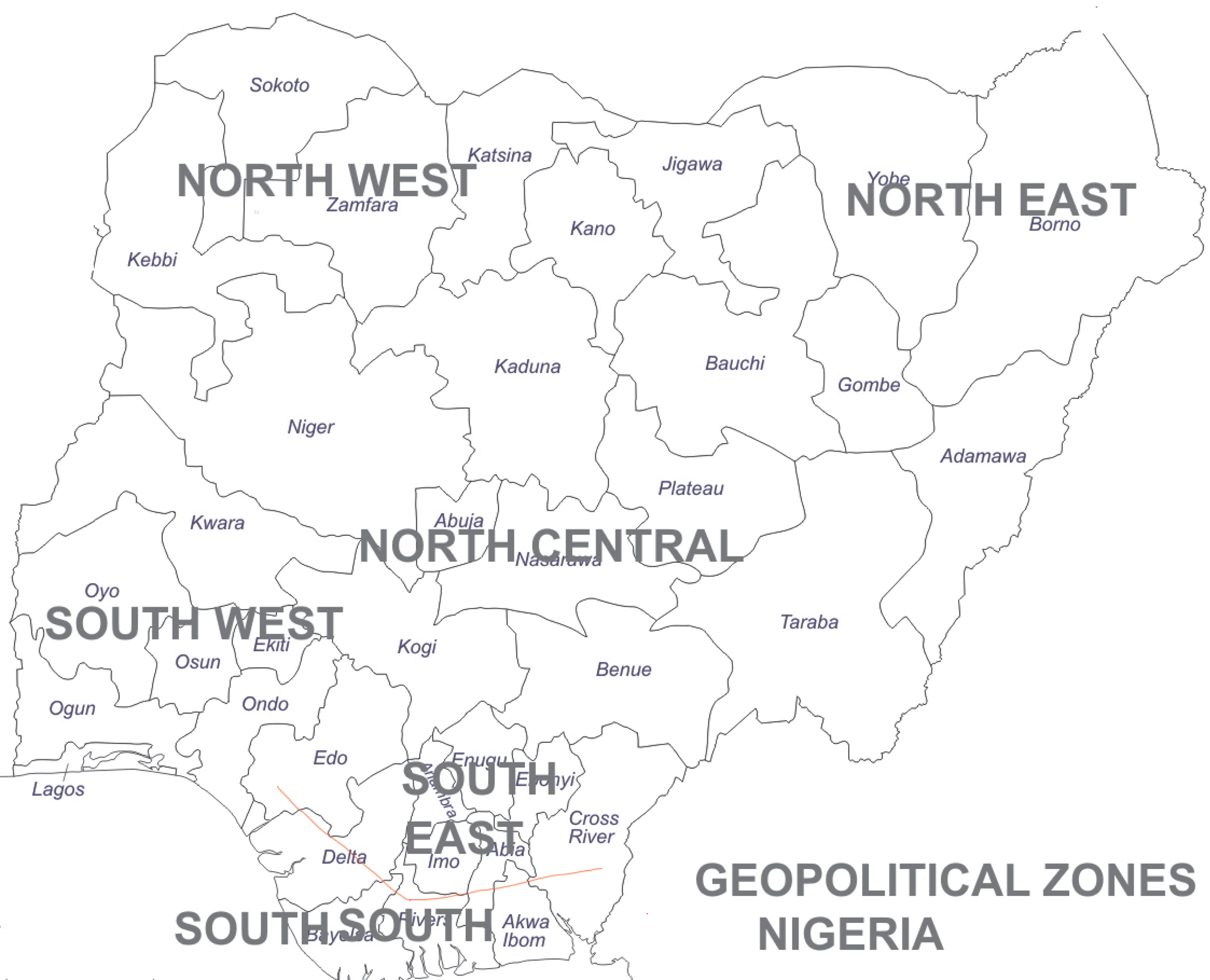

Supplement: Additional file 1: — Study regions and state. (PDF 373 kb) [file 12889_2015_2155_MOESM1_ESM.pdf]
