# Supplementary material for: Partner HIV serostatus disclosure and determinants of serodiscordance among prevention of mother to child transmission clients in Nigeria
Source: BMC Public Health. 2015 Aug 28;15:827. doi: 10.1186/s12889-015-2155-x (PMC4551711; doi:10.1186/s12889-015-2155-x)
Supplement: Additional file 2: — HIVQual sample size determination chart based on 95 % CI. (PDF 173 kb) [file 12889_2015_2155_MOESM2_ESM.pdf]

Additional file 2 HIVQUAL Sample Size Determination Chart based on a 95% CI.

Population Size	Sample Size for a 95% CI to have width of 0.16
Up to 20	All
30	26
40	32
50	38
60	43
70	48
80	53
90	57
100	61
101 – 119	67
120 -139	73
140 – 159	78
160 – 179	82
180 – 199	86
200 -249	94
250 -299	101
300 – 349	106
350 – 399	110
400 – 449	113
450 – 499	116
500 – 749	127
750 – 999	131
1000 – 4999	146
5000 or More	150
